# The External Validity of Randomized Controlled Trials of Hypertension within China: from the Perspective of Sample Representation

**DOI:** 10.1371/journal.pone.0082324

**Published:** 2013-12-06

**Authors:** Xin Zhang, Yuxia Wu, Deying Kang, Jialiang Wang, Qi Hong, Le Peng

**Affiliations:** 1 Department of Evidence-Based Medicine and Clinical Epidemiology, West China Hospital, Sichuan University, Chengdu, China; 2 Department of Internal Medicine, Mianyang people’s hospital, Mianyang City, China; Universidad Peruana Cayetano Heredia, Peru

## Abstract

**Objective:**

To explore external validity of randomized controlled trials (RCTs) of hypertension within China from the view of sample representation.

**Methods:**

Comprehensive literature searches were performed in Medline, Embase, Cochrane Central Register of Controlled Trials (CCTR) et al and advanced search strategies were used to locate hypertension RCTs as well as observational studies conducted in China during 1996 to 2009 synchronously. The risk of bias in RCTs and observational studies was assessed by two modified scales respectively, and then both types of studies with 3 or more grading scores were included for the purpose of evaluating of external validity. Following that the study characteristics relative to sample representation were extracted from RCTs and observational studies synchronously, and the later were taken as external references for validating sample representation of RCTs.

**Results:**

226 hypertension RCTs and 21 observational studies were included for final analysis. Comparing samples with observational studies, the mean age of samples within RCTs was 54.46 years, significantly lower than that of observational studies (66.35 years) (P=0.002). The average disease course in patients of RCTs was 3.89 years and grade III hypertensive patients accounted for 17%; both were lower than that of the observational studies (12.96 years, P<0.001; 34%, P=0.026 respectively). In addition, the proportions of patients with complications due to heart failure, stroke, diabetes, or coronary heart disease in RCTs were 8%, 5%, 12% and 11% correspondingly, all of which were significantly less than that of observational studies (11%, 18%, 17% and 29%).

**Conclusion:**

Sample characteristics within hypertension RCTs were significantly different from those in observational studies. The samples in most RCTs were under-represented. It’s feasible to take samples of observational studies as a mirror of the actual composition of hypertension patients in the real world, if the reporting of observational studies is abundant and available.

## Introduction

As the design and conduct has effectively eliminated the possibility of bias and confounding [1], randomized controlled trials (RCTs) having a favorable internal validity and being the gold standard for determining the effects of treatments, have been widely recognized in clinical researches [2-5]. Apart from the internal validity (i.e., whether the results suffer from systematic error) within RCTs, the external validity of RCTs needs to be emphasized too [6,7]; if RCTs were misused or the results from RCTs were irrelevant to the patients in a particular clinical setting [1,8,9], that may adversely affect to health care. Lack of external validity is frequently advocated as one of the obstacles to the translation of research evidence into clinical practice, which is why interventions found to be effective in clinical trials and recommended in guidelines are underused in clinical practice [1,10,11]. However, in comparison to internal validity, the external validity was easily neglected in clinical trials [6,9,10,12-14]; in addition, the assessment of the external validity is a complex reflection, studying how external validity assessments are also challenging. As currently, there is no consensus about how to assess the external validity of RCTs [9,15]. Some previous studies have highlighted somewhat potential determinants of external validity [9,14]; for example, strict eligibility criteria can limit the external validity of RCTs. A previous study indicated that fewer than 10% of patients with hypertension are managed in hospital clinics, and this group will differ from those managed in primary care [14]. However, external validity cannot be easily formalized [9] as the baseline clinical characteristics recorded often say very little about the real composition of the trial population. Easy to be quantified and reported abundantly, the sample representation is often used as an important indicator to assess external validity [16]; but, the lack of reference is frequently advocated as one of the obstacles to explore sample representation of RCTs. As few observational studies enrolled participants with stringent eligibility criteria, samples within observational studies were more likely representative, by which they could be candidate references for mirroring the real composition of patients in clinical practice. Hypertension has become a serious burden disease in China [17,18]; although a great number of clinical trials on hypertension have been conducted within China, few studies were successful in developing as evidence based information and disseminating to patients under specific circumstances [18]. This study intends to explore the sample representation in hypertension RCTs by comparing with the sample characteristics within observational studies.

## Materials and Methods

### Search strategy and study selection

A comprehensive literature search was performed; literature databases included Medline (Ovid), Embase, Cochrane Central Register of Controlled Trials (CCTR, Ovid), Chinese biomedical literature database (CBM), China National Knowledge Infrastructure/China Academic Journals Full-text Database (CNKI) and Chinese scientific journals database (VIP). The Medical Subject Headings (MeSH) ‘hypertension’, ‘randomized controlled trial’, ‘controlled clinical trial’, ‘random allocation’, ‘cases series’ and ‘cohort study’ were used as English and corresponding Chinese search terms to identify studies from the aforementioned databases (January 1, 1996 to December 31, 2009). In addition, references from included articles, as well as articles citing included articles, were screened for inclusion. 

Two authors (ZX and WYX) screened the titles and abstracts to identify relevant studies. In cases of disagreement, consensus was achieved by discussion with the third author (KDY). Criteria for final inclusion of RCTs included: (1) drug therapy for primary hypertension, in which six kinds of anti-hypertension drugs recommended by WHO were included (ACEI, Angiotensin-Converting Enzyme Inhibitor; ARB, Angiotensin II Receptor Blocker; CCB, Calcium Channel Blocker; alpha-blocker; beta-blocker; Diuretics); (2) studies with a grading score equal to or greater than 3. Similar criteria for final inclusion of observational studies were set: (1) topics on managing primary hypertension, in which six kinds of anti-hypertension drugs recommended by WHO were included; (2) any types of design related to cases series and cohort studies; (3) studies with a grading score equal to or greater than 3. RCTs were excluded if they: (1) recruited patients with secondary hypertension; (2) were published as abstracts only; (3) reported partial data from multi-center research. Observational studies were also excluded if they: (1) recruited patients with secondary hypertension; (2) had a sample size of less than 30; or (3) published repetitively.

### Internal validity assessment

Two kinds of scales for assessing internal validity of RCTs and observational studies were modified from five available tools; these included two RCTs-based tools: the Jadad scale [19] and the evaluation criteria in Cochrane Review’s Handbook [20]; and three tools for observational studies, including the Critical Appraisal Skills Programme (CASP) [21], Newcastle-Ottawa Scale (NOS) [22] and ‘Validity Checklist for Appraising an Article on Observational Study’ [23]. The scale developed for RCTs includes five domains: randomization (0–2 points), allocation concealment (0–2 points), blinding (0–2 points), attrition (0–2 points) and baseline condition (0–1 points); the total score for a perfect RCT is 9. Additionally, another scale for observational studies was used; as judgments associated with assessing quality in observational studies are often complex; here, we address four key issues that arise in assessing risk of bias: diagnostic criteria (0–1 points), sample source (0–1 points), recruitment (0–1 points) and setting of research (0–1 points); if an observational study eliminated the possibility of bias and confounding effectively, it would receive a grade of 4 points. 

A pilot study was then performed to validate the two modified scales; the agreement for each item (‘yes’ scores vs. any other scores) and the whole tool was explained by the percentage of actual agreement as well as the Kappa coefficient. We adopted the Kappa values of <0 rates as less than chance agreement, 0.01–0.20 as slight agreement, 0.21–0.40 as fair agreement, 0.41–0.60 as moderate agreement, 0.61–0.80 as substantial agreement, and 0.81–0.99 as almost perfect agreement [24].We tested the coding framework of RCT through comparison with the Jadad scale [25] and the criterion validity of the tool was assessed through calculating correlation coefficients. 

All included articles were rated using the above modified scales by two authors (ZX, WYX). Frequent ongoing discussions among all authors regarding any queries were proceeded throughout the coding process. 

### Data abstraction for evaluating external validity

Information for evaluating external validity was extracted by a pre-developed form [23,26]. Two authors (ZX, WYX) abstracted data independently and any discrepancies were resolved by discussion. The data extract form includes 4 domains and 25 items. The domain of “source” has 5 items: region of trial setting, research setting, date of study, number of centers involved, funding source; domain of “subjects recruitment” includes 7 items: location, setting, method, duration of recruitment, number of eligible patients, number of patients not meeting inclusion criteria, number of patients refusing participation; domain of “baseline characteristics of subjects” has 8 items: sample size, source of patients, age, gender, diagnosis criteria, duration of disease, state of disease, complications; the last domain relates to patients importance outcomes, includes “effectiveness outcomes” and “adverse events” respectively.

### Statistical analysis

Data were analyzed using SPSS software, version 13.0 (SPSS, Chicago, IL) and MetaXL, version 1.3 (MetaXL, Brisbane,Australia). Descriptive statistics, such as rate and proportion were used for dichotomous data, and means ± SDs or median (range) for continuous data. Correlation coefficients were taken to validate criterion validity of the modified scales for internal validity. T-test, Mann-Whitney test and multiple linear regression were used to test sample representation in terms of the age, duration of disease and proportions of female, grade III hypertension and other main complications. Generic Inverse Variance (GIV) method [27] was used to synthesize rate and proportion statistics reported in observational studies. We also used the guidelines for inferential interpretation of the overlap of CIs between two independent group rates or means to identify statistically significant difference: P <0.05 when the proportion overlap of the 95% CIs is ≤0.50 and P <0.01 when the two CIs do not overlap, that is, when proportion overlap is about 0 or there is a positive gap [25]. All tests were two-sided and *P* values of 0.05 or less were considered to be of statistical significance.

## Results

### Flow of included studies

**Figure 1:**
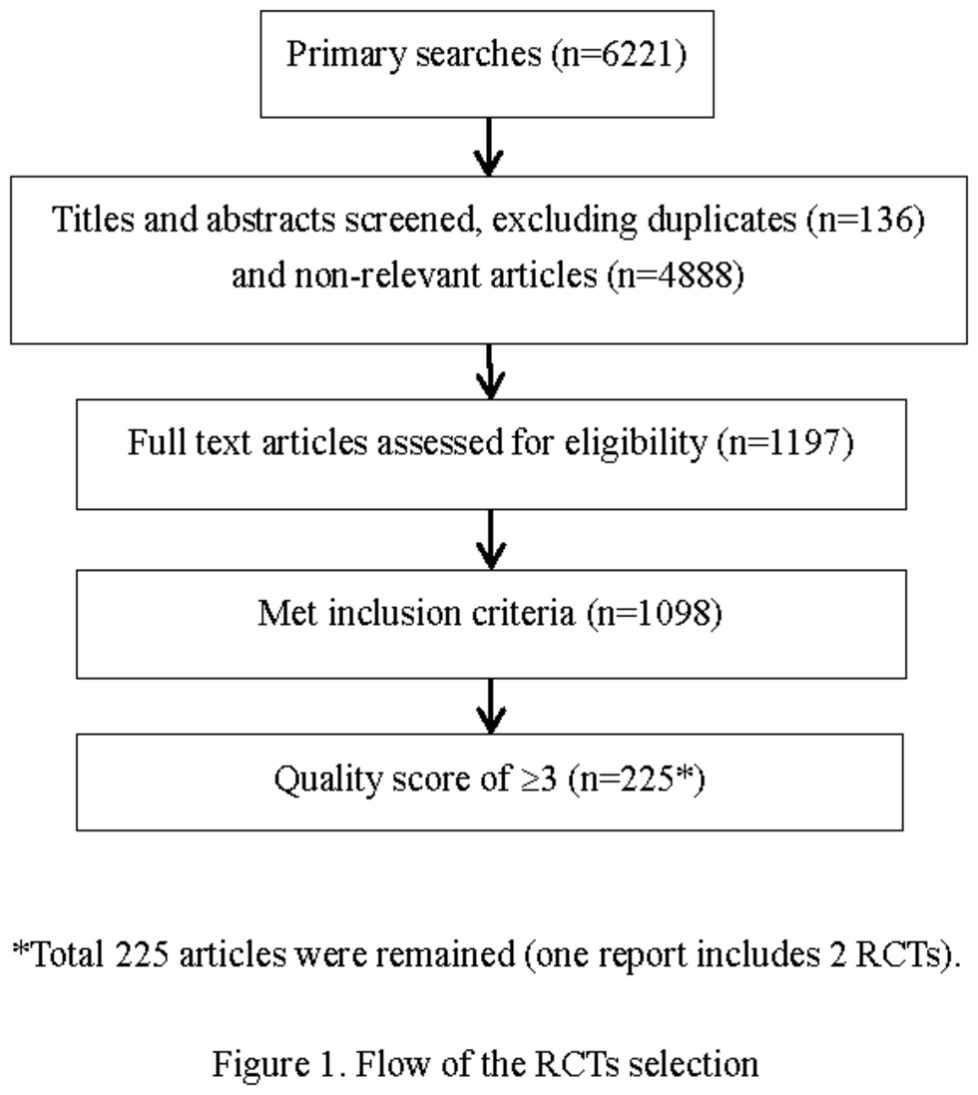
Flow of the RCTs selection.

 1197 RCTs were identified from the searches (excluding 136 duplicates and 4888 non-relevant articles), after that, 99 RCTs were excluded based on the inclusion criteria; finally, 225 RCTs with internal validity scores of ≥3 remained (Figure 1) 

**Figure 2:**
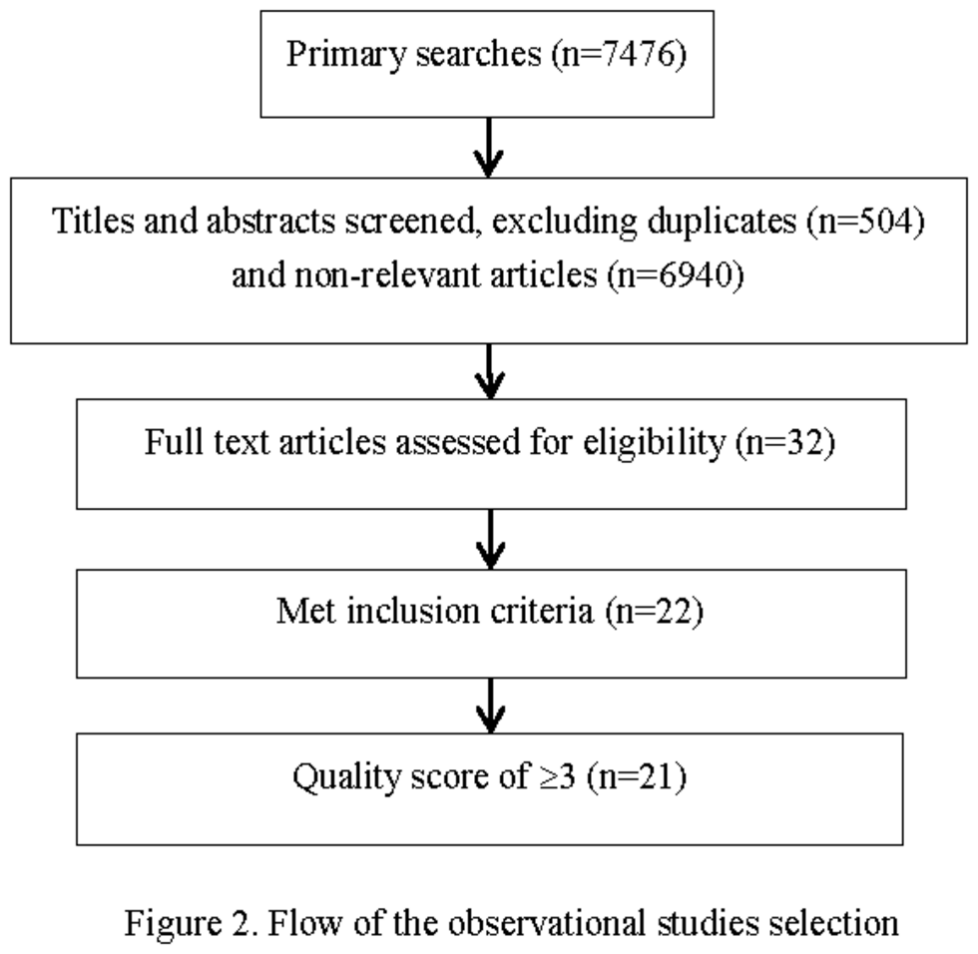
Flow of the observational studies selection.

Meanwhile, 32 observational studies were identified from the searches (excluding 504 duplicates and 6940 non-relevant articles), 10 observational studies were further excluded based on the inclusion criteria; 21 observational studies with quality scores of ≥ 3 were finally included (Figure 2)

 Clinical studies, either RCTs or observational studies, may suffer bias and confounding in their design or conduct, and incur additional risk of misleading results. Therefore, we take 3 as cut-off point for inclusion criterion of RCTs, which is equivalent to one third of total score of 9; as observational studies were more likely suffered bias and confounding than that of RCTs, means that strict eligibility criterion is needed, so we use 3 as the cut-off point for including observational studies, which is equivalent to the upper quartile of total score of 4.

### Validation of modified scales for internal validity

We selected 50 RCTs randomly using a computer-generated list to validate inter-rater agreement. The kappa between two assessors for the global assessment was 0.72 and the percentage of actual agreement was 76%（both P<0.001). (Table 1) 

**Table 1:** Assessment of the internal validity of selected 226 RCTs and agreements of inter-raters.

Item	Yes,n(%)	No,n(%)	Unclear,n(%)	Raters		P value
				Agreement	Kappa	
Randomization	48(21.2)	3(1.3)	175(77.4)	98%	0.90	0.001
Allocation concealment	14(6.2)	212(93.8)	0(0.0)	100%	—	—
Blinding	82(36.3)	73(32.3)	71(31.4)	96%	0.88	0.001
Attrition	16(7.1)	98(43.4)	112(49.6)	88%	0.63	0.001
Baseline condition	185(81.9)	41(18.1)	—	90%	0.80	0.001
Total	—	—	—	76%	0.72	0.001

Another 30 RCTs were randomly selected for validity evaluation. The total mean score was converted into the percentage of the maximum score for the modified scale, and the ICC against Jadad score was 0.84; that is, the results of the modified scale were highly convergent with the results of Jadad score. However, as the number of observational studies was limited, the validation procedure didn’t perform adequately. 

### Internal validity of included studies after applying two modified scales

Internal validity of the selected 1099 RCTs (one citation includes 2 RCTs) was assessed by applying the modified scale for RCTs, of those, 226 RCTs with a grade of equal to or greater than 3 points were included (Table 2) , the median grade of RCTs was 3, RCTs with a grading score equal to or greater than 7 only accounted for 3.1% (n=7); 22 observational studies met inclusion criteria were evaluated by applying the modified scale for OBSs, 21 OBSs with a grading score equal to or greater than 3 were included (Table 2), the median score was 4. 

**Table 2:** Grades of Internal validity for RCTs and observational studies.

Scores of grading	n	Percentage (%)	Cumulative percentage (%)	
RCT (n=226)				
9	1	0.4	0.4	
8	2	0.9	1.3	
7	4	1.8	3.1	
6	17	7.5	10.6	
5	27	11.9	22.6	
4	48	21.2	43.8	
3	127	56.2	100.0	
OBS (n=21)				
4	12	57.1	57.1	
3	9	42.9	100.0	

RCT, Randomized Controlled Trial; OBS, observational study.

### Comparisons of study characteristics between RCTs and observational studies

Study characteristics, like sample size, location of setting and class of hospital, sample source and diagnosis criteria, therapy regimen and type of drug, patient important outcomes, et al, meet the minimum requirements for comparison analysis because of adequate reporting either in RCTs or in observational studies.

#### Sample size

The medians of sample size were 99 (min-max: 29-1352, total=57813) and 360 (min-max: 73-5106, total=15789) respectively for included RCTs and observational studies; the sample size in RCTs was smaller in general than that of observational studies (P<0.001). 

#### Location of setting and hospital class

All included studies reported the research setting (location of setting and class of hospital); no significant discrepancy was observed in location of setting and hospital class (both P>0.05) (Table 3).

**Table 3:** Setting of research.

Study type	Location of setting, n (%)				Hospital class, n (%)	
	South China	North China	Both		Primary hospital	Secondary or tertiary hospitals
RCT (n=226)	106(46.9%)	92(40.7%)	28(12.4%)		181(80.0%)	45(20.0%)
OBS (n=21)	13(61.9%)	8(38.1%)	0(0.0%)		16(76.2%)	5(23.8%)
P value	0.17				0.67	

RCT, Randomized Controlled Trial; OBS, observational study.

#### Sample source and diagnosis criteria

Sample source was reported in 111(49.1%) RCTs and 18(85.7%) observational studies accordingly (Table 4); 73.9% (82/111) RCTs recruited outpatients, while none of the 18 observational studies were found to do so. Of observational studies, 12(57.1%) recruited inpatients consecutively (P=0.001). One hundred and twenty-five (55.3%) RCTs and all of the 21(100.0%) observational studies reported diagnosis criteria (Table 5); of those, the percentage which used “China’s criteria” accounted for 28.8% (36/125) of RCTs, while correspondingly, the percentage was 19.0% (4/21) in observational studies (P=0.03). 

**Table 4:** Sample source.

Study type	Sample source, n (%)			P value
	Outpatient	Inpatient	Both	
RCT (n=111)	82(73.9%)	7(6.3%)	22(19.8%)	0.001
OBS (n=18)	0(0.0%)	14(77.8%)	4(22.2%)	

RCT, Randomized Controlled Trial; OBS, observational study.

**Table 5:** Diagnosis criteria.

Study type	Diagnosis criteria, n (%)			P value
	WHO	China	Others	
RCT (n=125)	84(67.2%)	36(28.8%)	5(4.0%)	0.03
OBS (n=21)	13(61.9%)	4(19.0%)	4(19.0%)	

RCT, Randomized Controlled Trial; OBS, observational study.

#### Therapy regimen

In observational studies, CCB (Calcium Channel Blocker) was the most addressed drug, 23.81% hypertensive patients took CCB routinely; conversely, ARB (Angiotensin Ⅱ Receptor Blocker) was the most addressed drug in randomized controlled trials. None of the observational studies addressed alpha–blockers, while the percentage reached to 5.75% (13/226) in RCTs (P=0.536). (Table 6)

**Table 6:** Comparisons of drug and therapy regimen in two types of studies.

Therapy regimen	Drug types	Number of RCTs (%)	Number of OBSs (%)	Statistic	P value*
Single drug	Alpha-blocker	13(5.75)	0(0.0)	0.382	0.536
	Alpha, beta-blocker	12(5.31)	1(4.76)	─	1.000**^*#*^**
	Beta-blocker	19(8.41)	4(19.05)	1.470	0.225
	ACEI	29(12.83)	3(14.29)	─	0.741**^*#*^**
	ARB	59(26.11)	2(9.52)	2.841	0.092
	CCB	43(19.03)	5(23.81)	0.058	0.809
	Diuretics	8(3.84)	4(19.04)	6.924	0.009
Drug combination or compound preparation		43(19.03)	2(9.52)	0.614	0.433
Total		226	21	─	─

Abbreviations: RCT, Randomized Controlled Trial; OBS, observational study; ACEI, Angiotensin-Converting Enzyme Inhibitor; ARB, Angiotensin II Receptor Blocker; CCB, Calcium Channel Blocker.

*indicate χ^2^ test, # Fisher's Exact Test used.

Regarding therapy regimens, 19.03% (n=43) RCTs were designated to test drug combinations or compound preparations, while the proportion in observational studies was 9.52% (n=2). However, no statistical significance was found (P=0.433).

#### Patient important outcomes

The blood pressure change and effective rate of anti-hypertension were the most addressed primary outcomes among RCTs; while the secondary outcomes in RCTs varies considerably, including cardiovascular death, QOL, health economics, adverse events, compliance, as well as intermediate measures (such as left ventricular hypertrophy, renal function, vascular endothelial function, pulse wave velocity, new-onset diabetes, resistance ameliorating effect) . Significant discrepancy was observed in effective rate of anti-hypertension and adverse events (both P<0.001). Outcomes set in RCTs are seldom identical to those in observational studies. (Table 7)

**Table 7:** Comparisons of patient important outcomes in two types of studies.

Study type	Primary outcomes			Secondary outcomes										
				Intermediate measures						Cardiovascular death*	QOL scores	Health economics	Adverse events	Compliance
	Blood pressure change	Effective rate of anti-hypertension		Left ventricular hypertrophy	Renal function	Vascular endothelial function	Pulse wave velocity	New-onset diabetes	Resistance ameliorating effect					
RCT (n=226)	213(94.2%)	176(77.9%)		21(9.3%)	12(5.3%)	7(3.1%)	3(1.3%)	4(1.8%)	5(2.2%)	10(4.4%)	13(5.8%)	1(0.4%)	177(78.3%)	na
OBS (n=21)	na	7(33.3%)		na	na	na	na	na	na	1(4.8%)	na	na	1(4.8%)	2(9.5%)
P value**^*#*^**	na	<0.001		na	na	na	na	na	na	1.000	na	na	<0.001	na

RCT, Randomized Controlled Trial; OBS, observational study; QOL, quality of life; na, not available.

^*^ Cardiovascular death comprised fatal myocardial infarction, left ventricular failure, fatal and non-fatal stroke (excluding transient ischaemic attack), ruptured abdominal aortic aneurysm and cardiac arrhythmia.

^#^ χ^2^ test or Fisher's Exact Test.

### Comparisons between RCTs and observational studies in terms of sample representation

#### Duration of disease

The duration of disease was presented in 42.5% of RCTs as well as 42.9% of observational studies (Table 8). Of those, the average disease course in patients of RCTs was significantly lower than that of observational studies (3.89±4.39 vs. 12.96±4.49, P<0.001)

**Table 8:** Meta-analysis of age and disease course reported in RCTs and observational studies.

Study type	Number of studies	Means	SD	Mean difference	95%CI *	P value
Age(yrs)						
RCT	167	54.46	6.34	-11.89	-18.66 ^~^-5.13	0.002
OBS	19	66.35	13.91			
Duration of disease (months)						
RCT	96	3.89	4.39	-9.07	-12.57 ^~^-5.56	<0.001
OBS	9	12.96	4.49			

RCT, Randomized Controlled Trial; OBS, observational study; SD, Standard Deviation.

^*^ The confidence interval of the difference.

#### Grade III hypertension

Proportion of grade III hypertension is presented in Table 9. Patients with grade III hypertension in RCTs were significantly underrepresented in comparison with observational studies, with overall proportions of 0.17 (95%CI: 0.09 to 0.28) and 0.34 (95%CI: 0.27 to 0.42) respectively (P=0.026).

**Table 9:** Meta-analysis of gender, state of disease and complications reported in RCTs and observational studies.

	Items	Number of studies	Total cases	Analysed cases	Overall proportion*	95%CI	Heterogeneity		P value**^*#*^**
							I^2^ (%)	Q test	
Proportion of female patients									
	RCT	200	54202	23578	0.41	0.40~0.42	87.79	1629.95	0.426
	OBS	19	15015	6358	0.39	0.36~0.42	91.34	207.78	
Proportion of grade III hypertension patients									
	RCT	4	441	85	0.17	0.09~0.28	85.14	20.19	0.026
	OBS	13	6754	2314	0.34	0.27~0.42	97.47	474.57	
Proportions of complications									
Heart failure									
	RCT	2	329	24	0.08	0.00~0.25	91.21	11.38	0.505
	OBS	6	6068	1604	0.11	0.02~0.23	98.27	289.03	
Stroke									
	RCT	6	17315	2633	0.05	0.00~0.14	99.62	1300.23	0.018
	OBS	13	12526	2844	0.18	0.10~0.27	99.22	1544.59	
Diabetes									
	RCT	15	15806	2656	0.12	0.07~0.18	96.16	364.43	0.141
	OBS	17	13753	2821	0.17	0.13~0.22	97.89	757.32	
CHD									
	RCT	13	18172	2046	0.11	0.07~0.16	97.09	411.88	0.125
	OBS	11	2742	1052	0.29	0.10~0.51	99.19	1238.46	
Renal insufficiency									
	RCT	1	100	33	0.33	0.24~0.43	—	—	<0.01
	OBS	10	7100	694	0.08	0.05~0.11	94.76	171.85	

RCT, Randomized Controlled Trial; OBS, observational study; ACEI, Angiotensin-Converting Enzyme Inhibitor; ARB, Angiotensin Ⅱ Receptor Blocker; CCB, Calcium Channel Blocker.

^*^ Random effects results.

^#^ Mann-Whitney U test.

#### Complications

Only 10.2% (n=23) RCTs presented the reporting of complications. Proportions of complications in RCTs were lower than those of observational studies in terms of heart failure (P=0.505), stroke (P=0.018), diabetes (P=0.141) and CHD (Coronary Atherosclerotic Heart Disease, P=0.125). However, the proportion of complicating renal insufficiency was higher than those patients from observational studies (P <0.01, zero overlap in two CIs).

#### Age, gender

Patient ages were presented in 73.9% of RCTs and 90.5% of observational studies (Table 8). Patients in RCTs were younger than those in observational studies: 54.46±6.34 versus 66.35±13.91 (P=0.002). Accordingly, the proportions of females are presented in Table 9. The proportions of females were 0.41(95%CI: 0.40 to 0.42) and 0.39 (95%CI:0.36 to 0.42) in 200 RCTs and 19 observational studies respectively (P=0.426).

Multiple linear regressions were further used to explore impact factors of age and gender underrepresentation, but only study type had statistical significance (both P<0.05). Similar analyses in terms of duration of disease, proportion of grade III hypertension and proportion of complication didn’t perform adequately due to the limited number of studies. (Table 10)

**Table 10:** Multiple linear regression in terms of age and gender underrepresentation.

	Unstandardized Coefficients		Standardized Coefficients (Beta)	t	P value	95% CI for B
	B	SE				
Age						
Constant	38.128	3.118	─	12.230	<0.001	31.980~44.276
Study type	13.006	1.960	0.428	6.636	<0.001	9.141~16.871
Gender	8.136	4.950	0.106	1.644	0.102	-1.625~17.896
Gender						
Constant	0.384	0.052	─	7.391	<0.001	0.282~0.487
Study type	-0.067	0.030	-0.169	-2.198	0.029	-0.127~-0.007
Age	0.002	0.001	0.126	1.644	0.102	0.000~0.004

t, t-value; CI, confidence interval; SE,standard error.

## Discussion

Therapeutic efficacy is often studied with observational surveys in clinical practice of patients whose treatments were selected non-experimentally. Observational studies have several advantages over randomized controlled trials (including lower cost, greater timeliness, and a broader range of patients). An important advantage of the expanded observational study is its ability to estimate treatment effects in this broader spectrum of clinical practice. In this study, we attempt to use samples from observational studies of hypertension in China to create references which mirror hypertension patients in the real world. There are several interesting findings in our study. Firstly, the characteristics of RCTs on hypertension were significantly different from observational studies in terms of sample size, sample source, diagnosis criteria, frequency of diuretics used and types of medicine. Insufficient trial size may cause over-homogenous patients to be enrolled; simultaneously, it confers insufficient power for the statistical test employed, the failure to attain a level of statistical significance does not necessarily mean that the two treatments being compared are identical [28]. In comparison to inpatients, outpatients may have mild hypertension, short disease duration and even different therapy; if too many outpatients are recruited in hypertension RCTs, it’s easy to get overestimated effects.

Secondly, samples in RCTs were underrepresented in terms of the elderly, disease course, grade III hypertension patients and complications. Patients in RCTs were more likely young, having short duration of disease, as well as lower proportions of concurrent stroke and renal insufficiency than those in actual clinical settings. Due to the discrepancy in clinical characteristics, clinical manifestations and treatments among different age hypertension patients are also disparate different. Including insufficient elderly patients in RCTs, on other words, the lack of efficacy and safety information on elderly people, will directly limit the application and generalization of trial results to such spectrums of patients. RCTs tend to include less serious or shorter disease duration patients, who generally response well to drugs and are less likely to suffer severe side effects or adverse events, making it easier to get beneficial results. However, side effects or adverse event rates may appear to rebound when the intervention is applied in routine clinical practice. With regards to medicine, Angiotension Conversion Enzyme Inhibitor (ACEI) and Angiotensin II Receptor Blocker (ARB) are recommended by China Guideline for hypertension prevention and control[18] for hypertension patients complicated with diabetes or renal insufficiency; however, most of the 88 RCTs excluded diabetic patients (n=73, 83.0%) and renal insufficiency (n=87,98.9%). Beta-blocker and ACEI were recommended for hypertension patients complicated with CHD or heart failure [29]; among the 48 available RCTs, 15 (31.2%) studies excluded CHD patients and 26 (54.2%) studies excluded heart failure patients. Ruling out patients with complications excessively in trials will directly weaken the sample representation, leading to the overestimation of intervention effects; that is, the conclusion may be valid only to the sample population, but not be applicable to patients in the real world. 

There are several limitations in our study. First, we assume that these cohorts represented the "real world" in China but they may be not either due to publication bias, the ideal reference to reflect patients in the "real world" come from nationwide large-scale survey, however, such survey is very difficult to perform due to financial, political or technique barriers. Second, as the reporting quality of the included original studies (either RCTs or observational studies), were not good enough, much information related to external validity was not reported or was reported insufficiently, making it hard to analyze the factors related to sample representation thoroughly, such as patients enrollment information (those who didn't fit the inclusion criteria, those who fit but refused to participate, and those who were finally enrolled in the trial). Incidentally, more than half (58.8%) of the RCTs did not report disease course of included participants, and only 23 (10.2%) RCTs described complications of patients. Though the inclusion and exclusion criteria for patients were prior set, it’s still unclear about patients’ characteristics and limit to apply the trial results to patients in real world. Therefore, there is marked room for improving quality of the reporting in RCTs, especially at the respects related to external validity. Third, high quality observational studies were insufficient to make-up external references, as only 21 studies were identified in this study; caution is needed to use those synthesized results as substitutes of patients in routine clinical practice. Case reports by nature have one person in them, while case series we refer to is a design to study only patients exposed to the interventions, both types raise serious questions about false positive results caused by chance if sample size is less than 30 cases. Additionally, the design of case control study is not really representative of the general population and would not serve as reasonable "gold standard" for comparison to any RCT for external validity. Such types of observational studies were excluded. Another potential limitation needs to be addressed too, that is, a considerable amount of issues and multiple comparisons being involved in our study, those issues may be hard to follow and multiple comparisons without correction may lead to false positive findings, that is, positive results may be caused by chance. 

Moreover, heterogeneity existed in most meta-analyses but cannot be explained fully by the differences in patients’ age, sample source, class of hospitals, or sample size; sources of heterogeneity need to be investigated in further researches. 

## Conclusion

The samples within hypertension RCTs in China are underrepresented in terms of elderly patients, patients with long disease course, patients with complications and grade III hypertension patients. Although observational studies are frequently performed as a substitute for the randomized clinical trial, the evidence from such surveys is frequently not convincing. Taking samples of observational studies to make-up of patients in the real world is somewhat feasible; however, more studies are needed to demonstrate the validity of our results and their generalizability. There is also marked room for improving quality of the reporting either in RCTs or in observational studies.
